# Physical activity, neuropsychiatric symptoms, and physical function in nursing home residents: the HUNT 70+ study

**DOI:** 10.1186/s11556-025-00389-4

**Published:** 2025-11-17

**Authors:** Stine Øverengen Trollebø, Karen Sverdrup, Atle Skjelbred, Kristin Taraldsen, Ellen Marie Bardal, Nina Skjæret-Maroni

**Affiliations:** 1 Department of Neuromedicine and Movement Science, Faculty of Medicine and Health Science, Norwegian University of Science and Technology, Trondheim, Norway; 2 Norwegian National Centre for Ageing and Health, Oslo, Norway; 3 Department of Geriatric Medicine, Oslo University Hospital, Oslo, Norway; 4 Department of Rehabilitation Science and Health Technology, Faculty of Health Sciences, OsloMet – Oslo Metropolitan University, Oslo, Norway

**Keywords:** Nursing home, Physical activity, Neuropsychiatric symptoms, Physical function, HUNT

## Abstract

**Background:**

As life expectancy increases, the incidence of age-related chronic health conditions and functional decline rises, increasing the need for institutional long-term care such as nursing homes. In Norway, 84% of nursing home residents have mild cognitive impairment or dementia. This population is characterized by a high burden of neuropsychiatric symptoms (NPS), which contribute to limited physical activity (PA) and a largely sedentary lifestyle. Regular PA is essential for maintaining physical function (PF) and independence, yet nursing home residents spend most of their time being sedentary. The present study aims to explore sensor-based measured PA patterns and investigate how PA is associated with NPS and PF in nursing home residents.

**Methods:**

This cross-sectional study used data from the fourth wave of the population-based Trøndelag Health Study 70-years-and-older cohort (HUNT4 70+), including activity data from a total of 163 nursing home residents. PA was measured using two accelerometers placed on the lower back and thigh. PA patterns were described through the activity types walking, standing, sitting, and lying, activity bouts, and transitions. NPS was assessed using the Nursing Home Version of the Neuropsychiatric Inventory (NPI-NH), and PF using the Short Physical Performance Battery (SPPB). All outcome variables were grouped based on cognitive impairment and dementia severity, set by clinical experts using the DSM-5 diagnostic criteria and the Clinical Dementia Rating (CDR) scale.

**Results:**

Participants’ mean age was 87.8 years, and 62% were female. With a daily average of 17.6 min walking, 1.1 h standing, 9.9 h sitting, and 12.7 h lying, our nursing home residents spent approximately 94% of the day being sedentary. Walking and standing appeared predominantly in bouts under 10 min across cognitive impairment and dementia severity. No association was found between time spent walking, standing, sitting, lying and transitions from sedentary behavior to activity with NPS. Time spent walking, standing, lying and transitions from sedentary behavior to activity was significantly associated with PF.

**Conclusions:**

PA mainly occurred in bouts shorter than 10 min and sitting and lying accounted for most of the daily behavior. No association was detected between time spent in different activities and NPS. Although time spent walking and standing was limited in all groups, it showed an association with PF. This may suggest that even small amounts of PA play a significant role in maintaining or improving physical capabilities in nursing home residents.

**Supplementary Information:**

The online version contains supplementary material available at 10.1186/s11556-025-00389-4.

## Background

As life expectancy continues to rise, a growing number of older adults are expected to face age-related health challenges, increasing the demand for comprehensive care [1]. Regular physical activity (PA) is essential for older adults to prevent disease, preserve physical function (PF), and the ability to perform activities of daily living (ADL) such as dressing, eating, and self-care [2–4]. Sustaining PF through regular PA can prevent functional decline, reduce the risk of falls and healthcare needs, and protect against adverse outcomes like increased morbidity and mortality [5–8].

PA plays a crucial role in maintaining independence, also among older adults with comprehensive care needs [9]. However, research has shown that 94% of older adults in institutional long-term care, e.g., nursing home care, spend the majority of their time sedentary [10]. This sedentary behavior can accelerate cognitive decline, increase dependence, reduce social interaction, and contribute to a greater burden of neuropsychiatric symptoms (NPS) in individuals with cognitive impairment and dementia [11, 12].

NPS refers to a range of behavioral and psychological changes, including agitation, aggression, irritability, euphoria, apathy, depression, anxiety, delusions, and hallucinations, that occur during the natural course of dementia [13, 14]. Approximately 80% of nursing home residents with dementia experience at least one clinically significant NPS [15]. Non-pharmacological interventions are recommended as the first-line treatment for managing NPS, with research suggesting that PA can reduce NPS through enhanced mood regulation and neuroplasticity [16–19]. However, few studies have investigated the relationship between PA, NPS, and PF using sensor-based measures. The use of sensor-based measures of PA may provide a more accurate description of daily PA behavior in nursing home residents. Further, PA constitutes multiple complex behaviors. Recent research advocates for a more comprehensive assessment that goes beyond intensity, including PA patterns such as timing, frequency, duration, and transitions between activities [20]. This detailed description of daily physical behavior has yet to be described in nursing home residents with cognitive impairment and dementia. The aim of this study was to (1) describe daily PA patterns including time, frequency and duration (bouts), and transitions across cognitive impairment and dementia severity in nursing home residents, and to (2) investigate the association between time spent walking, standing, sitting, and lying along with transitions from sedentary behavior to activity with NPS and PF.

## Methods

### Study population

The study population includes nursing home residents who participated in the fourth wave of the population-based study Trøndelag Health Study 70-years-and-older cohort (HUNT4 70+) [21]. In HUNT4 70+ (2017–2019), a district in the city of Trondheim was included (Trondheim 70+) to cover the representation of a larger city. The participants in HUNT4 70 + were aged 70 and older and underwent an age-specific clinical examination in addition to the overall HUNT protocol [13]. Special efforts were made to include nursing home residents, with ambulatory teams conducting assessments directly within nursing homes. HUNT4 70 + included residents from multiple nursing homes, and while all residents were eligible to participate, no information on the specific wards or unit types (e.g., open versus closed/secured wards) in which participants resided was available.

For the current study, we included nursing home residents with complete and valid activity data from a minimum of 24 consecutive hours, including both day- and night-time. Residents who removed their sensors before reaching 24 consecutive hours of wear time were excluded. Additionally, inclusion in this study required an assessment of participants’ cognitive function determined by two clinical experts. In total, 163 participants met the inclusion criteria and were included in this study.

### Ethics

All participants gave a broad informed written consent prior to participation in HUNT4 70 + and HUNT4 Trondheim 70+. If the participants were unable to consent themselves, their closest proxy was asked to consent on their behalf. The present study was approved by the Regional Committee for Medical and Health Research Ethics in Norway (REK Southeast C 729845).

### Measures and data analysis

PA was assessed using two accelerometers (Axivity AX3, Newcastle, UK), attached to the lower back and thigh of the participants. Detailed information about the placement and configuration of the sensors is provided elsewhere [22]. The raw accelerometer data was analyzed using the Human Activity Recognition 70+ (HAR70+) model, classifying the activity types walking, standing, sitting, lying, running, and cycling over 60-second windows and in a 24-hour perspective [22]. Visual inspection of the raw acceleration data was conducted in group by multiple raters to assess classifications of activities with known weaknesses in the HAR70 + model, e.g. misclassification of walking with a walker as cycling [22]. During visual inspection, all classified cycling appeared to be walking and were therefore changed accordingly. This misclassification likely occurred since walking with a walker generates accelerometer features that resembles those of cycling, particularly when participants lean forward on the walker and exhibit a low stepping frequency [22]. No accelerometer data was classified as running.

The following outcome variables were calculated to describe PA patterns using in-house software developed in Python (Python Software Foundation, DE, USA) and include:

1. Mean time (in minutes) spent walking, standing, sitting, and lying, calculated across all valid days.
2. Bouts of walking, standing, sitting, and lying, calculated by identifying continuous periods of these activities sustained without interruption. Walking and standing bouts were classified into bout lengths of 1–2 min, 2–3 min, 3–10 min, 10–30 min, and > 30 min, while sitting and lying bouts were classified into bout lengths of 1–5 min, 5–10 min, 10–30 min, 30–60 min, and > 60 min. The cut-offs were determined based on visual inspection, where narrow intervals were decided useful for the short walking and standing bouts, whereas broader categories were appropriate for longer bouts typically observed during sitting and lying activities.
3. Transitions from sedentary behavior to activity calculated by collating transitions from lying to standing, lying to walking, sitting to standing, and sitting to walking, and expressed as daily mean.

The open-access code used to calculate PA patterns is available at https://github.com/AtleSkjelbred/axivity-pp-nh.

NPS was assessed by a caregiver at the nursing home [23], using the Nursing Home Version of the Neuropsychiatric Inventory (NPI-NH), which evaluates the frequency and severity of 12 different psychiatric or behavioral symptoms. NPI-NH is a reliable and valid instrument for assessing psychiatric symptoms and behavioral disturbances in nursing home residents, giving a total NPI-score ranging from 0 to 144 [24, 25]. In analyses, NPS was clustered into the subsyndromes agitation (agitation/aggression, disinhibition, irritability, 0–36), psychosis (delusions and hallucinations, 0–24), affective symptoms (depression and anxiety, 0–24), and apathy (apathy, 0–12) based on previous research on nursing home residents [25]. Higher scores within each subsyndrome indicated more severe NPS [26, 27].

PF was assessed using the Short Physical Performance Battery (SPPB), which includes a timed hierarchic balance test, a 4-meter gait speed test, and a repeated sit-to-stand test [28]. The balance test consisted of three different standing positions: feet together, semi-tandem, and tandem stance. Gait speed was measured twice over a 4-meter distance using the best time, and the repeated sit-to-stand was measured as the ability to rise from a chair five times at a maximal rate. Each test was scored separately on a scale of 0 to 4, and the scores were then summed to yield a total score ranging from 0 to 12, with higher scores indicating better PF. All residents were included in the SPPB assessment regardless of mobility status. Residents who attempted but were unable to complete parts of the SPPB test battery were assigned a score of 0 for any sub-tests they could not perform.

Two clinical experts set an independent diagnosis of mild cognitive impairment and dementia according to the DSM-5 diagnostic criteria [13, 29]. The detailed process and diagnostic criteria have been described elsewhere [13]. For participants diagnosed with dementia, dementia severity was categorized using the Clinical Dementia Rating (CDR) scale, which characterizes six domains of cognitive and functional performance [30]. The Norwegian CDR is reliable and validated for assessment of dementia severity in nursing home residents [31–33]. Sum of boxes across the six domains provides a total score ranging from 0 to 18. A score of 0 indicates no dementia, scores between 0.5 and 4.0 represent questionable to very mild dementia, 4.5 to 9.0 indicate mild dementia, 9.5 to 15.5 correspond to moderate dementia, and scores from 16.0 to 18.0 reflect severe dementia. Due to the small sample size diagnosed with no cognitive impairment (*n* = 4), these were combined with individuals diagnosed with mild cognitive impairment. Hence, in the current study, participants were classified into the following groups: (1) no cognitive impairment and mild cognitive impairment consisting of amnestic and non-amnestic mild cognitive impairment, (2) mild dementia, (3) moderate dementia, and (4) severe dementia.

Height and weight were measured as part of the main HUNT4 clinical examination, and body mass index (BMI) was calculated by dividing the weight in kilograms by the square of the height in meters (kg/m²).

### Statistical analysis

All outcome variables in this study were categorized by cognitive impairment and dementia severity. Participant characteristics were reported as number (n), mean (SD), and range (min/max) based on cognitive impairment and dementia severity. A normality test was performed, and as all data were non-normally distributed based on visual inspection of histograms and Q-Q plots, Kruskal-Wallis test followed by Dunn’s test was performed on continuous variables to compare group-by-group differences between cognitive impairment and dementia severity. Differences between age and sex were also assessed using the Kruskal-Wallis test.

To describe PA patterns, including time, bouts, and transitions across the four levels of cognitive impairment and dementia severity, the Kruskal-Wallis test was conducted, followed by Dunn’s test. The same test was applied to check for differences in time spent in different activities between age and sex. Before determining associations between time spent in different activities and transitions from sedentary behavior to activity with NPS and PF, scatter plots and correlation coefficients were used to assess variable relationships. Correlations ranged from moderate (PA and PF = 0.56, and transitions and PF = 0.63) to low (e.g., PA and agitation = 0.05). As assumptions were met, a generalized linear model (GLM) with gamma distribution and log link was applied to examine associations in a single model.

Outliers were retained in the analysis, as they reflect genuine variability rather than data entry errors or measurement artifacts. No statistical transformations or exclusions were applied, ensuring that the full range of the population’s heterogeneity is represented. A 0.05 significance level and 95% confidence intervals were used. Analyses were performed using Stata version 18 (18.0, Stata-Corp LLC, College Station, TX).

## Results

### Participant characteristics

A total of 163 nursing home residents with a mean age of 87.8 years (range: 70–103, 62% female) were included in this study. Of these residents, 25 were diagnosed with no or mild cognitive impairment, 73 with mild dementia, 47 with moderate dementia, and 18 with severe dementia. The mean total NPI score across all participants was 10.74, with sub scores of 4.05 for agitation, 1.58 for psychosis, 2.22 for affective symptoms, and 0.76 for apathy. Significant differences were observed for all NPS scores across cognitive impairment and dementia severity (Table 1).

The overall mean SPPB score was 2.05 (range: 0–11), with individuals with severe dementia showing significantly lower scores compared to those with no/mild cognitive impairment and less severe dementia. No significant differences were found in age, sex, or BMI between cognitive impairment and dementia severity (Table 1).

**Table 1:** Participant characteristics by cognitive impairment and dementia severity

	Total (*n* = 163)	No/mild cognitive impairment (*n* = 25	Mild dementia (*n* = 73)	Moderate dementia (*n* = 47)	Severe dementia (*n* = 18)	Group diff.*
**Age**,** y**						No sig. diff
Mean (SD)	87.74 (7.51)	88.50 (8.88)	88.68 (7.12)	85.99 (7.43)	87.41 (6.97)	
Range (min/max)	70–103	70–100	73–103	73–100	70–96	
**Sex**,** No. (%)**						No sig. diff
Female	101 (62)	14 (56)	50 (68)	24 (51)	13 (72)	
Male	62 (38)	11 (44)	23 (32)	23 (49)	5 (28)	
**BMI (kg/m²)**						No sig. diff
Mean (SD)	25.99 (5.53)	24.40 (4.46)	26.66 (5.83)	26.45 (5.69)	23.61 (4.84)	
Range (min/max)	16–52	17–36	16–52	19–37	19–33	
**NPI Agitation**						< 0.05 ^b, c, d, e^
Mean (SD)	4.05 (6.65)	1.04 (2.99)	2.58 (4.78)	7.04 (8.94)	6.44 (6.49)	
Range (min/max)	0–36	0–12	0–22	0–36	0–21	
**NPI Psychosis**						< 0.05 ^b, d^
Mean (SD)	1.58 (3.61)	0.68 (2.43)	0.96 (3.38)	3.04 (4.31)	1.56 (2.89)	
Range (min/max)	0–24	0–12	0–24	0–24	0–9	
**NPI Affective symptoms**						< 0.05 ^b, d^
Mean (SD)	2.22 (3.63)	0.92 (2.48)	1.58 (2.88)	3.38 (4.35)	3.61 (4.53)	
Range (min/max)	0–15	0–12	0–10	0–15	0–14	
**NPI Apathy**						< 0.05 ^c, e^
Mean (SD)	0.76 (2.0)	0.08 (0.28)	0.44 (1.36)	1.04 (2.42)	2.28 (3.21)	
Range (min/max)	0–12	0–1	0–6	0–9	0–12	
**Total NPI**						< 0.05 ^b, c, d, e^
Mean (SD)	10.74 (13.86)	3.68 (7.08)	7.25 (10.13)	18.06 (18.05)	15.56 (12.12)	
Range (min/max)	0–98	0–30	0–46	0–98	0–47	
**SPPB**						< 0.05 ^c, e, f^
Mean (SD)	2.05 (2.44)	1.84 (1.89)	2.68 (2.83)	1.85 (2.08)	0.28 (0.75)	
Range (min/max)	0–11	0–7	0–11	0–7	0–3	

Group diff.* Kruskal-Wallis and Dunn’s test to check for significant differences between groups (p < 0.05) illustrated through

*SD* Standard deviation, *Range* Min/max, *BMI* Body Mass Index, *NPI Agitation* Neuropsychiatric symptom cluster of agitation/aggression, disinhibition, irritability (0–36), *NPI Psychosis* Neuropsychiatric symptom cluster of delusions and hallucinations (0–24), *NPI Affective symptoms* Neuropsychiatric symptom cluster of depression and anxiety (0–24), *NPI Apathy* Neuropsychiatric symptom cluster of apathy (0–12), *Total NPI* Total Neuropsychiatric symtpom score (0–144), *SPPB* Short Physical Performance Battery (0–12)

^a^No/mild cognitive impairment vs. Mild dementia, ^b^No/mild cognitive impairment vs. Moderate dementia, ^c^No/mild cognitive impairment vs. Severe dementia, ^d^Mild dementia vs. Moderate dementia, ^e^Mild dementia vs. Severe dementia, ^f^Moderate dementia vs. Severe dementia

### Physical activity patterns

**Fig. 1:**
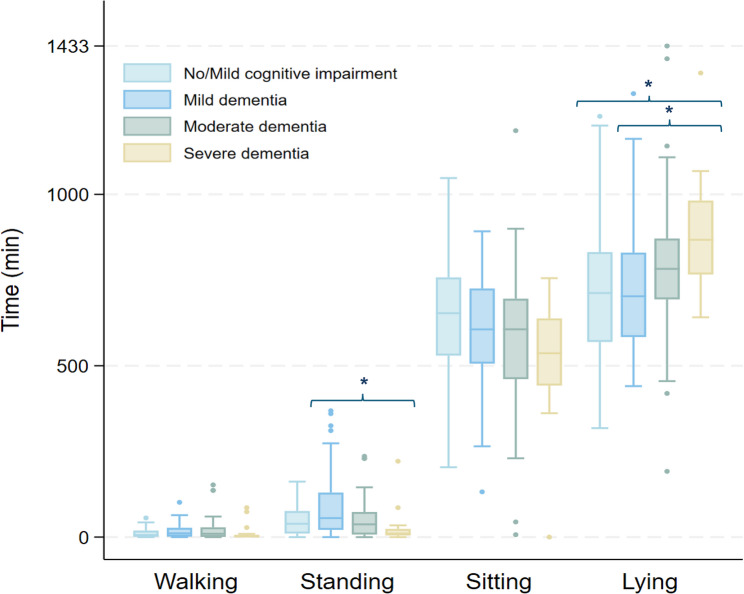
Distribution of mean daily minutes in walking, standing, sitting, and lying across cognitive impairment and dementia severity. * Indicates statistically significant group differences (Kruskal-Wallis test)

Nursing home residents spent a daily average of 17.6 min walking, 1.1 h standing, 9.9 h sitting, and 12.7 h lying, which corresponds to 94% of their day spent in sedentary behavior. Individuals with severe dementia spent significantly less time standing per day compared to those with mild dementia (27.34 min vs. 89.31 min, *p* < 0.05) (Fig. 1, Additional file 1). Individuals with severe dementia spent significantly more time lying per day compared to those with no/mild cognitive impairment (14.68 h vs. 12.25 h, *p* < 0.05) and mild dementia (14.68 h vs. 12.10 h, *p* < 0.05) (Fig. 1). No differences were detected in time spent in different activities between age (all p’s > 0.05) or sex (all p’s > 0.05).

On average, nursing home residents had 22 daily transitions from sedentary behavior (lying or sitting) to activity (standing or walking) (range: 0–73, SD:17.30) (see Additional file 2). Individuals with no/mild cognitive impairment had an average of 22 transitions per day (range: 0–73, SD: 17.34), those with mild dementia averaged 27 transitions (range: 0–60, SD: 16.93), individuals with moderate dementia averaged 21 transitions (range: 0–66, SD: 17.41), and those with severe dementia had on average 10 transitions per day (range: 0–46, SD: 12.50). Transitions from sedentary behavior to activity differed between dementia severity, showing a decreasing trend in mean transitions from mild to severe dementia.

**Fig. 2:**
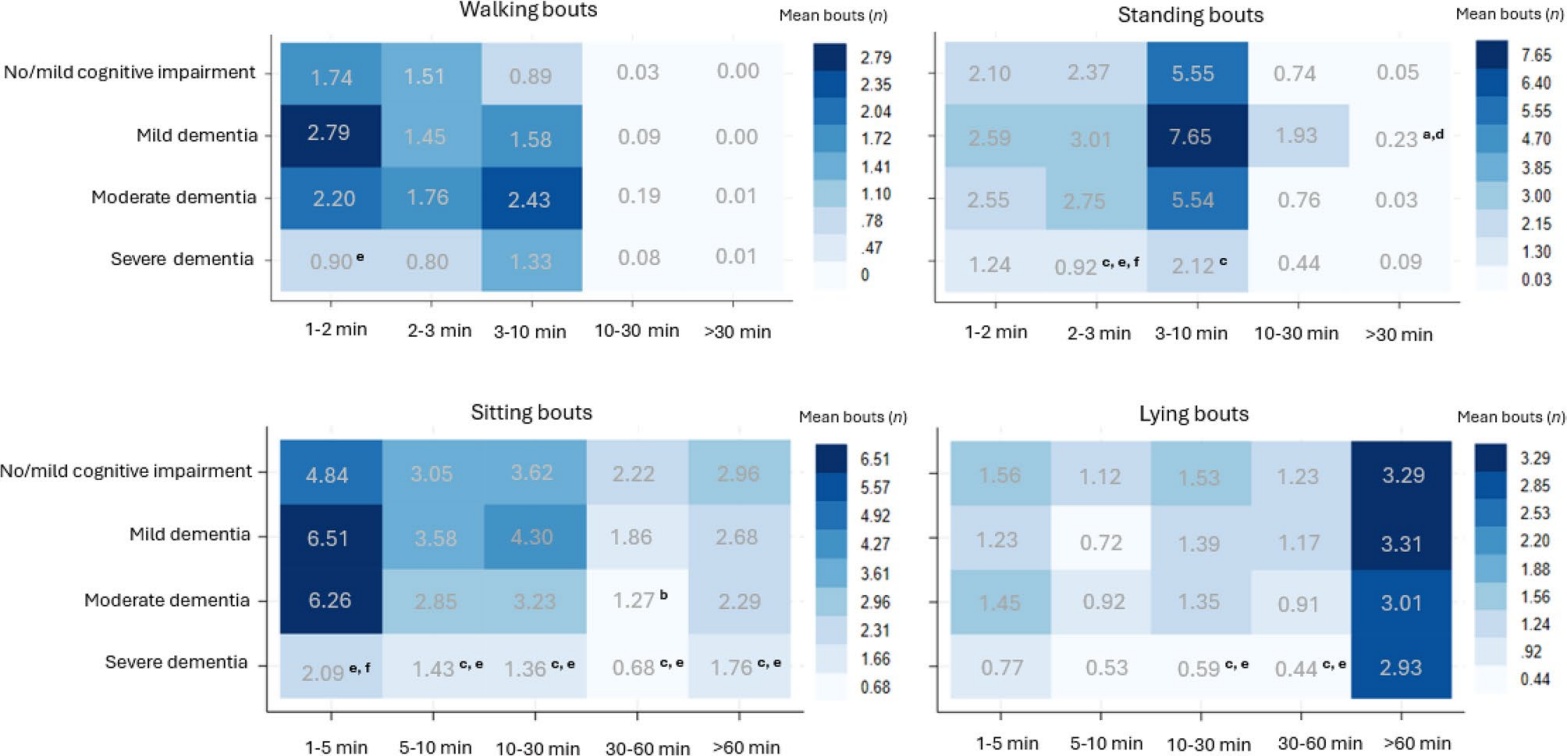
The frequency (n) of mean daily bouts by activity type across bout duration, and cognitive impairment and dementia severity. Number indicates mean daily bouts. Letters indicate significant group differences (*p* < 0.05) identified using Kruskal-Wallis and Dunn’s test: ^a^ No/mild cognitive impairment vs. Mild dementia, ^b^ No/mild cognitive impairment vs. Moderate dementia, ^c^ No/mild cognitive impairment vs. Severe dementia, ^d^ Mild dementia vs. Moderate dementia, ^e^ Mild dementia vs. Severe dementia, ^f^ Moderate dementia vs. Severe dementia

As seen in Fig. 2, the highest frequency of walking and standing bouts appeared in bout lengths under 10 min, in sitting bouts lasting from 1 to 5 min, and lying bouts exceeding 60 min across cognitive impairment and dementia severity (see Additional file 3). Individuals with severe dementia exhibited significantly fewer walking bouts lasting 1–2 min compared to those with mild dementia. They also had fewer standing bouts lasting 2–3 min compared to all other groups, and fewer 3–10-minute standing bouts compared to individuals with no/mild cognitive impairment. In terms of sitting behavior, those with severe dementia had significantly fewer bouts lasting 1–5 min than individuals with mild and moderate dementia, and fewer bouts lasting 5–10, 30–60, and over 60 min compared to those with no/mild cognitive impairment and mild dementia. Additionally, they showed a lower frequency of lying bouts lasting 10–30 and 30–60 min compared to individuals with no/mild cognitive impairment. 

Significant differences were also detected among individuals with no/mild cognitive impairment and moderate dementia, showing fewer standing bouts exceeding 30 min compared to individuals with mild dementia. Moreover, individuals with moderate dementia exhibited fewer 30–60-minute sitting bouts than those with no/mild cognitive impairment.

### Association of PA with NPS and PF

No association was detected between time spent in the different activities and NPS (all p’s > 0.05). Time in walking, standing and transitions from sedentary behavior to activity was positively associated, and lying was negatively associated with PF (all p’s < 0.05). The exponentiated coefficients indicate that one minute increase in average daily walking (0.35) corresponded to a 42% rise in PF, one additional minute in average daily standing (0.25) and one additional transition from sedentary behavior to activity (0.25) corresponded to a 28% increase in PF, while one minute increase in average daily lying (− 0.02) corresponded to a 2% decrease in PF (Table 2).

**Table 2:** Associations between time spent in different activities and transitions from sedentary behavior to activity with NPS and PF

	Coefficient	Std. error	*P*-value	95% CI
**Average daily walking (min)**				
NPI Agitation	0.02	0.02	0.417	−0.02 to 0.06
NPI Psychosis	−0.00	0.04	0.956	−0.08 to 0.08
NPI Affective symptoms	0.01	0.04	0.841	−0.07 to 0.09
NPI Apathy	−0.08	0.06	0.202	−0.20 to −0.4
SPPB	0.35	0.07	0.000	0.21 to 0.48
Cognitive impairment and dementia severity	0.22	0.16	0.156	−0.09 to 0.53
**Average daily standing (min)**				
NPI Agitation	0.02	0.02	0.394	−0.02 to 0.06
NPI Psychosis	−0.02	0.03	0.628	−0.09 to 0.05
NPI Affective symptoms	0.02	0.04	0.665	−0.06 to 0.09
NPI Apathy	−0.01	0.06	0.066	−0.21 to 0.01
SPPB	0.25	0.06	0.000	0.14 to 0.36
Cognitive impairment and dementia severity	−0.20	0.16	0.222	−0.05 to 0.12
**Average daily sitting (min)**				
NPI Agitation	−0.00	0.00	0.812	−0.01 to 0.01
NPI Psychosis	−0.00	0.01	0.847	−0.02 to 0.02
NPI Affective symptoms	−0.01	0.01	0.218	−0.02 to 0.01
NPI Apathy	−0.00	0.01	0.779	−0.03 to 0.02
SPPB	−0.01	0.01	0.400	−0.03 to 0.01
Cognitive impairment and dementia severity	−0.05	0.03	0.097	−0.01 to 0.01
**Average daily lying (min)**				
NPI Agitation	−0.00	0.00	0.944	−0.01 to 0.01
NPI Psychosis	−0.00	0.01	0.831	−0.01 to 0.01
NPI Affective symptoms	0.01	0.01	0.175	−0.00 to 0.02
NPI Apathy	0.01	0.01	0.378	−0.01 to 0.03
SPPB	−0.02	0.03	0.025	−0.04 to −0.00
Cognitive impairment and dementia severity	0.04	0.03	0.116	−0.01 to 0.09
**Transitions from sedentary behavior to activity (n)**				
NPI Agitation	0.02	0.12	0.158	−0.01 to 0.04
NPI Psychosis	−0.01	0.02	0.802	−0.05 to 0.04
NPI Affective symptoms	0.03	0.02	0.178	−0.01 to 0.08
NPI Apathy	−0.08	0.04	0.027	−0.15 to −0.01
SPPB	0.25	0.04	0.000	0.18 to 0.32
Cognitive impairment and dementia severity	−0.11	0.09	0.263	−0.29 to 0.08

*NPI Agitation* Neuropsychiatric symptom cluster of agitation/aggression, disinhibition, irritability (0–36), *NPI Psychosis *Neuropsychiatric symptom cluster of delusions and hallucinations (0–24), *NPI Affective symptoms* Neuropsychiatric symptom cluster of depression and anxiety (0–24), *NPI Apathy * Neuropsychiatric symptom cluster of apathy (0–12), *SPPB* Short Physical Performance Battery (0–12), *Cognitive impairment and dementia severity* Assessed using DSM-5 diagnostic criteria and Clinical Dementia Rating (CDR) Scale

## Discussion

This study aimed to explore physical activity patterns in nursing home residents across cognitive impairment and dementia severity. Further, we investigated the association between time spent in different activity types with neuropsychiatric symptoms and physical function.

Our results on PA patterns showed that nursing home residents spent a daily average of 17.6 min walking and 1.1 h standing and had on average 22 transitions from sedentary behavior to activity a day. Nursing home residents exhibited fewer walking and standing bouts with durations exceeding 10 min, had mostly sitting bouts lasting from 1 to 5 min, and lying bouts that exceeded 60 min. No association was found between time spent walking, standing, sitting, lying and transitions from sedentary behavior to activity with NPS, but an association was detected between time spent walking, standing, lying and transitions from sedentary behavior to activity with PF.

### Physical activity patterns

Our study found that sitting and lying were the dominant types of daily behavior among nursing home residents, corresponding to spending 94% of their day being sedentary. As a dementia diagnosis can lead to restlessness and nighttime activity [34], we examined PA from a 24-hour perspective to provide a more comprehensive picture of PA in nursing homes [35]. However, the total amount of time spent in sedentary behavior in our study aligns with previous research on self-reported PA, also suggesting that 94% of nursing home residents spend their time sitting or lying, even if they are capable of participating in independent activities [36].

Moreover, our study shows that nursing home residents, regardless of cognitive impairment and dementia severity, predominantly engage in walking bouts lasting less than 10 min. As suggested by Del Din et al. (2016), short walking bouts are likely to represent indoor walking, while longer durations are more common for outdoor walking [37]. It is likely to assume that most nursing home residents spend most of their time indoors, where their movements are limited to short distances within hallways, rooms, and common areas, leading to shorter bouts of walking. However, since our bout categorization grouped walking and standing bouts into durations from 3 to 10 min, we cannot determine whether these activities lasted closer to 3 min or 10. This uncertainty reflects the broader issue of inconsistency in classifying PA bouts, potentially causing variations in reported activity patterns and impacting the overall comparability of studies [38].

Furthermore, our study supports previous findings suggesting that standing constitutes the primary component of upright activity [39]. Standing breaks within walking bouts are common, making prolonged, uninterrupted walking less frequent [40]. It is reasonable to believe that this pattern is similar within nursing homes, where such breaks may occur due to interactions with healthcare workers, the need for stability, or during personal care. The relatively high frequency of standing bouts under 10 min may reflect the fragmented nature of daily routines in nursing homes, where residents frequently stop their movement due to assistance needs, environmental constraints, or limited mobility. Another possible explanation for the high number of standing bouts is the activity classification method used in this study. As activities are predicted in 60-second epochs, brief walking episodes may be misclassified as standing if standing makes up most of the 60-second epoch. This is likely to happen among nursing home residents, as previous research has demonstrated that most of the walking activity in older adults occurs in bouts lasting less than a minute, and that frequent standing occurs within walking bouts [39, 41, 42].

Our findings further indicate that individuals with severe dementia engage in less standing compared to those with no/mild cognitive impairment and less severe dementia. This is also reflected in the lower number of daily transitions observed in this group. The frequency of walking and standing bouts supports this pattern, as individuals with severe dementia exhibited fewer short-duration bouts, suggesting reduced variation in their daily PA. Reduced variation in activity patterns has previously been associated with cognitive impairment, and is corroborated in our study [43]. The World Health Organization emphasizes that engaging in any level of PA is better than none, highlighting the importance of even short bouts of PA for health outcomes [44]. This might be particularly relevant for nursing home residents with severe dementia, as implementing brief activity periods could have beneficial implications for their health [45–47].

However, the observed significant difference in walking bouts between the mild and severe dementia group is likely influenced by the relatively large sample size in the mild dementia group, which increases the statistical power to detect group differences. In contrast, when comparing groups with more similar sample sizes, such as those with no/mild cognitive impairment group and severe dementia, the study may have insufficient power to detect differences of similar magnitude. This suggests that the lack of significant group differences in these analysis does not necessarily indicate the absence of meaningful differences but are rather influenced by small and uneven group sizes. Moreover, nursing home residents with no/mild cognitive impairment are likely admitted primarily due to physical impairments, which may actually account for a greater similarity to individuals with severe dementia compared to those with mild or moderate dementia [48].

The higher PA levels observed in groups with less severe dementia may be due to better cognitive functioning, as previous research has demonstrated that severe dementia is a predictor of functional decline in nursing home residents [49–51]. Further, approximately 70% of individuals with dementia experience apathy, which may lead to reduced motivation and general interest in participation [52]. Interestingly, our study showed no differences in daily PA between sex or age. This is contrary to previous studies that have demonstrated that men are more physically active than women and that PA decreases with increasing age [39]. The lack of relationship between sex and age may be attributed to a floor effect, whereby the overall low levels of PA across all participants limit the variation necessary to detect group differences. Moreover, a generally poor physical or cognitive condition is often the primary reason for admission to a nursing home today, which may overshadow any variations typically associated with age or gender.

### Association of PA with NPS and PF

Our study did not find an association between daily PA and NPS in nursing home residents. This contrasts with Ishimaru et al. [53], who reported that agitation and aggression were linked to increased PA in individuals with advanced dementia, supporting previous findings that agitation tends to intensify with disease progression and is often expressed through increased activity [54, 55]. Similarly, our results differ from the review by Scherder et al. [12], who suggested that inactivity may contribute to increased agitation in dementia. One possible explanation for this discrepancy is the relatively low levels of NPS observed in our sample, which may have limited the ability to detect associations. Our study population included relatively few individuals with moderate to severe dementia, whereas both Ishimaru et al. [53] and Scherder et al. [12] examined participants with a dementia diagnosis, including more advanced stages. In contrast, we used data from the HUNT study, which recruited the general nursing home population rather than specifically targeting individuals with dementia. A further consideration for some of the observed findings is the potential presence of selection bias in our study sample. Our study only included nursing home residents that wore activity sensors, and we lack information on those who did not, and the reason for it. This issue is also highly relevant for the discussion of the feasibility of using sensors in this population, as it raises questions about how representative the included sample is of the overall nursing home population. Consequently, our sample may exhibit lower overall levels of NPS, which could partly explain why no association between NPS and PA was detected.

In addition, factors such as medical comorbidities, medication use, or institutional routines may place a stronger influence on NPS than PA, potentially obscuring any direct relationship. In nursing home settings, daily life is typically structured around shared routines such as personal care and meals, which all residents participate in regardless of functional status. Prior research indicates that environmental factors play an increasingly important role in shaping PA with advancing age [56]. Thus, daily PA among nursing home residents may be more determined by environmental conditions and staff support, than by the actual cognitive abilities of the resident.

Our study found a strong association between daily average walking, standing, lying and transitions from sedentary behavior to activity with PF. Each additional minute of walking and standing per day was linked to a 42% and 28% higher SPPB score, respectively. Likewise, each additional transition from sedentary behavior to activity was associated with a 28% higher score. In contrast, each additional minute spent lying down was linked to a 2% lower SPPB score. These results may indicate a positive effect of walking and standing on PF, whereas prolonged lying may have a negative impact. Although the amount of walking and standing performed by the included nursing home population was generally low, these results emphasize that even small amounts of daily PA can make a meaningful difference in preventing further physical decline. Moreover, the observed association between the number of daily transitions from sedentary behavior to activity and PF aligns with previous research indicating that fragmented PA patterns characterized by frequent transitions between sedentary and active states are linked to better PF outcomes in older adults [57, 58]. These findings suggest that not only the total amount of PA but also the pattern, including frequency of transitions, may play a crucial role in maintaining or enhancing PF in older adults. Therefore, supporting residents to engage in regular movement throughout the day is important for retaining their independence. However, this study is a cross-sectional study that does not allow for a determination of causality or prediction with respect to the relationship between PA and PF. It is therefore unclear whether improved PF results from higher activity levels or if higher activity levels are a result of better PF.

### Strengths and limitations

A key strength of this study was the relatively large sample with activity data from nursing home residents. Collecting data from individuals with cognitive impairment and dementia using wearable technology is often a challenge, leading to the exclusion of this population in many studies. Moreover, this study included activity monitoring of both nursing home residents who were ambulatory and those who were not, along with SPPB data from residents regardless of mobility status. Residents who attempted but were unable to complete the SPPB-test battery were assigned a score of 0 on any sub-tests they could not perform, ensuring their inclusion in the analysis. This is contrary to previous research that has only included ambulant residents and will thereby contribute to increasing the representativeness of the current study [59]. The study also comes with several limitations. Firstly, the cross-sectional design of our study does not allow for any interpretation of causality. Moreover, we did not include any information on which ward the participants belonged to (e.g., open versus closed/secured). Differences in ward layout, staffing, and daily routines could potentially affect residents’ opportunities for PA, and the lack of these details limits our ability to account for such contextual influences in the analyses. This study did not account for the use of medications, including sedatives, antipsychotics, or antidepressants, which represents an important limitation, as these medications can substantially influence both PA and NPS. Our study sample was restricted to nursing home residents who wore activity sensors, potentially introducing selection bias. We had no information on residents who did not wear sensors or reasons for not participating which may limit the generalizability of our findings. Further, this lack of information may limit our ability to determine whether our sample represents the healthiest segment of nursing home residents, potentially with better PF and lower levels of NPS, which could have influenced the overall findings. In addition, group sizes varied, and results from smaller groups should be interpreted with caution, as they are more vulnerable to random variations.

## Conclusion

Our study showed that nursing home residents spend approximately 94% of their time being sedentary, and that physical activity mainly occurs in bouts of less than 10 min. Nursing home residents with severe dementia tend to engage in less PA compared to those with less severe dementia. No association was detected between time spent in the different activities and NPS, but our findings indicate that even small amounts of walking and standing along with transitions from sedentary behavior to activity are strongly associated with PF in nursing home residents. These results highlight the importance of implementing simple, practical strategies within nursing homes to encourage residents to increase their daily activity, thereby supporting the maintenance or enhancement of PF. However, little is known about how to effectively promote PA among nursing home residents, underscoring the need for further investigation on how to make feasible and tailored interventions in a nursing home setting.

## Supplementary Information

Supplementary Material 1.

Supplementary Material 2.

Supplementary Material 3.

## Data Availability

Data is not publicly available. Data may be obtained from the HUNT database (https://www.ntnu.edu/hunt).
