# Supplementary material for: Physical activity, neuropsychiatric symptoms, and physical function in nursing home residents: the HUNT 70+ study
Source: Eur Rev Aging Phys Act. 2025 Nov 17;22:23. doi: 10.1186/s11556-025-00389-4 (PMC12625522; doi:10.1186/s11556-025-00389-4)
Supplement: Supplementary file 1 — Supplementary Material 1. [file 11556_2025_389_MOESM1_ESM.pdf]

Additional file 1: Mean daily time in different activities across cognitive impairment and dementia severity

	No/mild cognitive impairment (*n=25*)	Mild dementia (*n=73*)	Moderate dementia (*n=47*)	Severe dementia (*n=18*)	Group diff.*
**Time spent walking (min)**					
Mean (SD)	12.38 (14.86)	17.71 (19.60)	22.42 (35.91)	12.42 (25.54)	No sig. diff
Range	0-56	0-102	0-152	0-86	
**Time spent standing (min)**					
Mean (SD)	50.79 (46.60)	89.31 (93.00)	52.21 (60.70)	27.34 (52.48)	<.05 [e]
Range	0-162	0-369	0-236	0-221	
**Time spent sitting (min)**					
Mean (SD)	641.70 (222.34)	606.83 (161.30)	570.21 (205.68)	519.74 (173.28)	No sig. diff
Range	204-1048	132-892	7-1186	0-756	
**Time spent lying (min)**					
Mean (SD)	735.12 (234.50)	726.15 (182.08)	795.16 (220.48)	880.51 (171.88)	<.05 [c, e]
Range	318-1228	441-1294	192-1433	641-1354	

SD: Standard deviation, Range=Min/max, Group diff.= Kruskal-Wallis and Dunn's test to check for significant differences between groups (p<0.05) illustrated through: a= No/mild cognitive impairment vs. Mild dementia, b= No/mild cognitive impairment vs. Moderate dementia, c= No/mild cognitive impairment vs. Severe dementia, d= Mild dementia vs. Moderate dementia, e= Mild dementia vs. Severe dementia, f= Moderate dementia vs. Severe dementia.*
