# Supplementary material for: Physical activity, neuropsychiatric symptoms, and physical function in nursing home residents: the HUNT 70+ study
Source: Eur Rev Aging Phys Act. 2025 Nov 17;22:23. doi: 10.1186/s11556-025-00389-4 (PMC12625522; doi:10.1186/s11556-025-00389-4)
Supplement: Supplementary file 2 — Supplementary Material 2. [file 11556_2025_389_MOESM2_ESM.pdf]

Additional file 2: Mean daily transitions from sedentary behavior to activity across cognitive impairment and dementia severity

	**No/mild cognitive impairment (*n=25*)**	**Mild dementia (*n=73*)**	**Moderate dementia (*n=47*)**	**Severe dementia (*n=18*)**	**Group diff.***
**Transitions from sedentary behavior to activity (n)**					
Mean (SD)	22.02 (17.34)	26.68 (16.93)	20.79 (17.41)	10.45 (12.50)	<.05 [e]
Range	0-73	0-60	0-66	0-46	

SD: Standard deviation, Range=Min/max, Group diff.= Kruskal-Wallis and Dunn's test to check for significant differences between groups (p<0.05) illustrated through: a= No/mild cognitive impairment vs. Mild dementia, b= No/mild cognitive impairment vs. Moderate dementia, c= No/mild cognitive impairment vs. Severe dementia, d= Mild dementia vs. Moderate dementia, e= Mild dementia vs. Severe dementia, f= Moderate dementia vs. Severe dementia.*
