# Supplementary material for: Physical activity, neuropsychiatric symptoms, and physical function in nursing home residents: the HUNT 70+ study
Source: Eur Rev Aging Phys Act. 2025 Nov 17;22:23. doi: 10.1186/s11556-025-00389-4 (PMC12625522; doi:10.1186/s11556-025-00389-4)
Supplement: Supplementary file 3 — Supplementary Material 3. [file 11556_2025_389_MOESM3_ESM.pdf]

Additional file 3: The frequency (n) of mean daily bouts by activity type across bout duration, and cognitive impairment and dementia severity.

	**No/mild cognitive impairment (*n=25*)**	**Mild dementia (*n=73*)**	**Moderate dementia (*n=47*)**	**Severe dementia (*n=18*)**	**Group diff.***
**Walking bouts (n)**					
1-2 min	1.74	2.79	2.20	0.90	<.05 [c, e]
2-3 min	1.51	1.45	1.76	0.80	No sig. diff
3-10 min	1.33	1.58	2.43	1.33	No sig. diff
10-30 min	0.03	0.09	0.19	0.08	No sig. diff
>30 min	0.00	0.00	0.01	0.01	No sig. diff
**Standing bouts (n)**					
1-2 min	2.10	2.59	2.55	1.24	No sig. diff
2-3 min	2.37	3.01	2.75	0.92	<.05 [c, e, f]
3-10 min	5.55	7.65	5.54	2.12	<.05 [e]
10-30 min	0.74	1.93	0.76	0.44	No sig. diff
>30 min	0.05	0.23	0.03	0.09	<.05 [a, d]
**Sitting bouts (n)**					No sig. diff
1-5 min	4.84	6.51	6.26	2.09	<.05 [e, d]
5-10 min	3.05	3.58	2.85	1.43	<.05 [c, e]
10-30 min	3.62	4.30	3.23	1.36	<.05 [c, e]
30-60 min	2.22	1.86	1.27	0.68	<.05 [b, c, e]
>60 min	2.96	2.68	2.29	1.76	<.05 [c, e]
**Lying bouts (n)**					
1-5 min	1.53	1.23	1.45	0.77	No sig. diff
5-10 min	1.12	0.72	0.92	0.53	No sig. diff
10-30 min	1.53	1.39	1.35	0.59	<.05 [c, e]
30-60 min	1.23	1.17	0.91	0.44	No sig. diff
>60 min	3.29	3.31	3.01	2.93	No sig. diff

n= Number of bouts, Group diff.= Kruskal-Wallis and Dunn's test to check for significant differences between groups (p<0.05) illustrated through: a= No/mild cognitive impairment vs. Mild dementia, b= No/mild cognitive impairment vs. Moderate dementia, c= No/mild cognitive impairment vs. Severe dementia, d= Mild dementia vs. Moderate dementia, e= Mild dementia vs. Severe dementia, f= Moderate dementia vs. Severe dementia.*
